# Fluorescence Ratiometric Properties Induced by Nanoparticle Plasmonics and Nanoscale Dye Dynamics

**DOI:** 10.1155/2013/624505

**Published:** 2013-05-27

**Authors:** Aron Hakonen

**Affiliations:** Department of Chemistry and Molecular Biology, University of Gothenburg, Kemivägen 10, 412 96 Gothenburg, Sweden

## Abstract

Nanoscale transport of merocyanine 540 within/near the plasmon field of gold nanoparticles was recognized as an effective inducer of single-excitation dual-emission ratiometric properties. With a high concentration of the signal transducer (ammonium), a 700% increase in fluorescence was observed at the new red-shifted emission maximum, compared to a nanoparticle free sensor membrane. A previously nonrecognized isosbestic point is demonstrated at 581.4 ± 0.1 nm. The mechanism can be utilized for enhanced and simplified ratiometric optical chemical sensors and potentially for thin film engineering to make solar cells more effective and stable by a broader and more regulated absorption.

## 1. Introduction

Currently metal nanoparticles, nanostructures, and plasmonics are of substantial scientific interest. Applications include chemical sensors, nanophotonics, and photovoltaics; for example, see [1–17]. 

 Already in 1957 the concept of surface plasmons was suggested by Ritchie in the form of “fast electrons” effectively absorbing resonance energy from nearby molecules [18]. Surface plasmons are in general described as oscillating electronic excitations near a metal surface and can be well characterized by classical electrodynamics [2, 19]. Enhanced fluorescence by plasmonic interactions owes in part or completely to the degeneration of fluorescence decays and the consequent increase of decay rates and decrease in fluorescence lifetime [19]. If the fluorescence emissions originating from the fluorophore or the metal surface are indistinguishable, therefore, the emitting complex (dye/metal surface) could more accurately be treated as an entity with the proposed name plasmophore [2]. Plasmophore fluorescence normally/always displays the fluorophores typical emission spectrum [2].

 Plasmonic enhancement of fluorescence is closely related to fluorescence quenching by metal surfaces and is in principle two opposites arising from the same physical feature that originates from the nanoparticle-fluorophore interactions. In essence, wavevector matching [2] for each explicit plasmophore event determines whether a photon will be emitted to far-field or be quenched [20]. Normally, at a few-nanometer distance, the metal is prone to quench the fluorescence, while at intermediate distances (approximately 5–50 nm) plasmophore emission is dominating, with an optimum typically in the 10–20 nm range; exact distances are physically dependent on the specific systems materials, geometry, and nanoscale.

 Optical chemical sensors have proven a potential for single-point measurements as well as for imaging purposes; for example, see [21–37]. Some optical chemical sensors have been based on the principles of coextraction of solvatochromic dyes [38–41]. A highly sensitive optical sensor for ammonium (1.7 nM detection limit) based on dynamic plasmon interactions in cooperation with a solvatochromic shift has been demonstrated [42] and has later been utilized for imaging of ammonium and ammonia release from biological tissues [20, 43]. The original concept of this sensing scheme involves a movement of the fluorophore toward the alkylthiol gold nanoparticles detained in the oily phase of a hydrogel/ether emulsion (1–6 *μ*m droplets) [42], with the ether droplets as containers for ensembles of the plasmophore complex [20]. These dual excitation ratiometric measurements are gained from several synergetic nanoparticle interactions. For example the ratiometric signal amplitude is increased by a surface plasmon enhancement which is gradually shifting to quenching as the fluorophore coextracts (in presence of analyte) and get closer to the nanoparticle surface [42]. Also, scattering enhancement of the longer wavelength emission has been proposed [42]. A smooth and practical square root diffusion consistent analyte dependence has been shown for the original dual-excitation dual-emission wavelength-sensing scheme [44].

 In present work a significantly altered fluorescence of the nanoparticle enhanced sensing scheme discussed above is demonstrated. The changes involves a charge transfer mechanism, which introduces several useful and interesting characteristics. 

## 2. Materials and Methods

GNPs came from Sigma-Aldrich and were of 10 nm particle size (~0.01% HAuCl_4_, ~0.75 A_520_ units/mL, 8.5–12.0 nm mean particle size (monodisperse)). 1-Dodecanethiol, nonactin, 2-(dodecyloxy) benzonitrile, tetrahydrofuran (THF), dimethyl sulfoxide (DMSO), merocyanine 540 (MC540), ammonium chloride, sodium chloride, and ethanol were all reagent grade and purchased from Sigma-Aldrich. The HN80 Hypan hydrogel came from Hymedix Inc., and MilliQ water (electric resistivity > 18 MΩ cm^−1^) was obtained from a Millipore water purification system. Stock solutions of dodecanethiol (10 mM in ethanol), MC540 (7 *μ*M in MilliQ water), ammonium chloride (2 mM in MilliQ), and sodium chloride (2 mM in MilliQ) were prepared.

 The alkanethiol monolayer on the gold nanoparticles was prepared as by Hakonen [42]. Dodecanethiol (250 *μ*L, 10 mM in ethanol) was mixed with 500 *μ*L of the GNP solution and allowed to form monolayers for 48 hours during intense shaking (1800 min^−1^). To eliminate GNPs with diminutive thiol monolayer, 75 *μ*L of ether (2-(dodecyloxy)benzonitrile) was added to the mixture to extract GNPs hydrophobic enough to prefer the fatty ether. The solution was vigorously shaken (2400 min^−1^) for ten minutes, after which it was allowed to phase separate for 48 hours. A three-phase system appeared, and the clear upper ether phase was removed carefully to be used in the sensors. 

 The sensing membranes were also prepared as by Hakonen [42]. HN80 hydrogel (69 mg) and the ionophore nonactin (2.8 mg) were weighed in a 5 mL glass vial, DMSO (2.1 mL) was added, and the vial was sealed. The sensor blend was stirred and heated to 150°C for ~30 min. THF (0.9 mL) was slowly added during continuing stirring (but not heating). Ether with and without GNPs was added in various amounts to produce membranes with different quantities of GNPs. The blank sensor was added to 6 *μ*L ether without GNPs, and sensor 1 was added to 2 *μ*L of ether with GNPs and 4 *μ*L without. Sensors 2 and 3 were added only to ether with nanoparticles, 6 and 30 *μ*L, respectively. The sensor cocktails (0.5 mL per sensor) were spread onto a transparency film (Corporate Express, code 608 83 29) and allowed to form emulsion overnight in a semiclosed container with an air humidity of ~60%. Access solvents were rinsed away with MilliQ. The sensors were stained by immersing in a 7 *μ*M solution of MC540 for six hours. Before fluorescence measurements, the sensing membranes were immersed in NaCl (2 mM) solution. Note that the only difference between the blank sensor and the other sensors is the addition of GNPs. 

 The fluorescence experiments were performed on a FluoroMax 3 instrument from Jobin Yvon (Horiba group). A standard plastic cuvette for fluorescence was used, and the sensing membranes were mounted at a 30° angle of incidence from the excitation source. The equilibrium time for both increases and decreases in ammonium concentration was 15 min. Excitation and emission spectra were collected with slit widths corresponding to 2 nm bandpass; integration times were set to 0.25 s and 1 nm steps. Solutions for the spectra were obtained by mixing appropriate amounts of ammonium chloride and sodium chloride stock solutions (for a constant ionic strength of 2 mM). 

## 3. Results and Discussion

**Figure 1:**
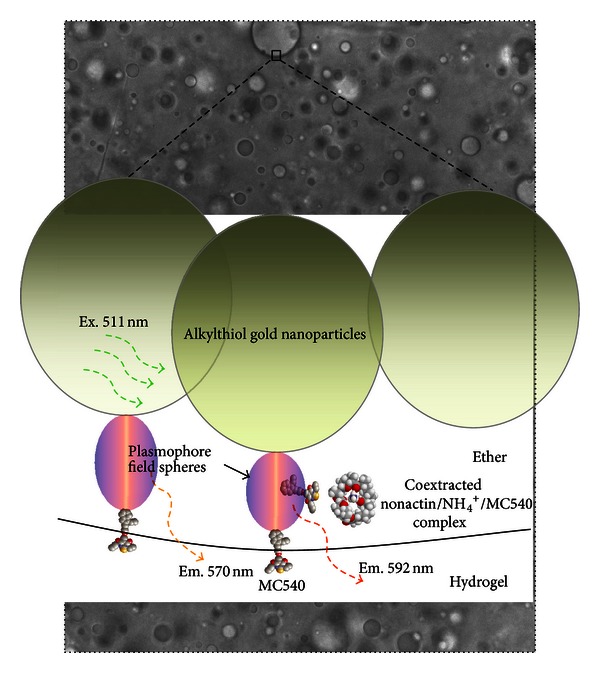
A small surface subsection of an emulsion droplet is viewed (the background is a microscopy image of the hydrogel/ether emulsion). One dye molecule has been coextracted into an imagined plasmophore field sphere acting as an effective FRET acceptor.

**Figure 2:**
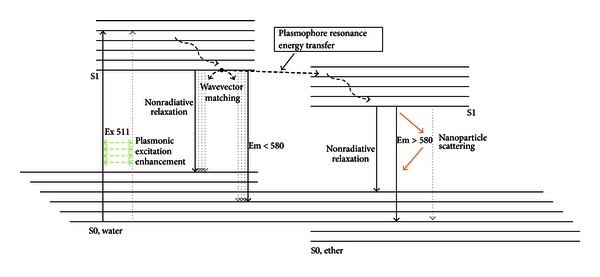
A proposed modified Jablonski diagram for the system. Dotted arrows indicate nanoparticle interactions.

In the present work, the concept of plasmophore resonance energy transfer by dye dynamics, at a liquid interface with nearby gold nanoparticles, is proposed as an efficient pathway for energy assimilation and charge transfer (Figure 1). The main electronic transitions of the plasmophore- (dye/metal-) decoupled dye assembly are summarized in Figure 2.

**Figure 3:**
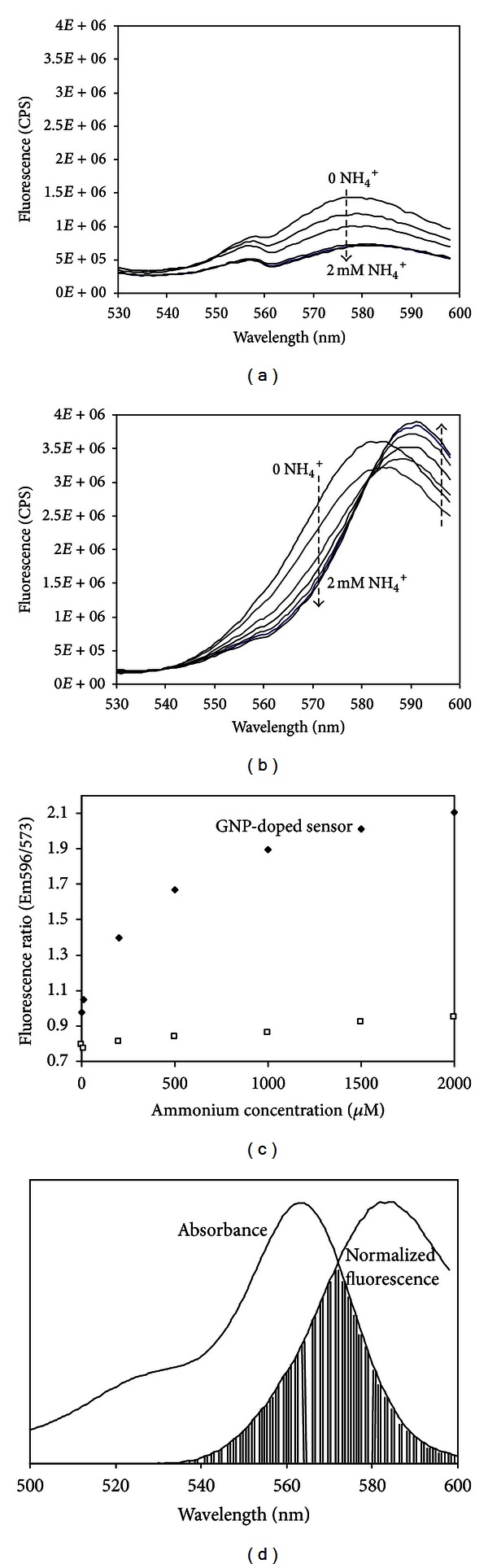
MC540 fluorescence at 511 nm excitation for an ammonium (concentrations 0, 10, 200, 500, 1000, 1500, and 2000 *μ*M) sensor without (a) and with (b) nanoparticles. (c) Calibration curves for sensors without (□) and with nanoparticles (*◆*). (d) Acceptor absorbance, donor fluorescence, and the FRET overlap peaking at 572.5 nm.

The significant change in fluorescence properties that the nanoparticles induce within the sensing membranes is demonstrated in Figures 3(a) and 3(b). In addition to the increased intensity, a shift in fluorescence is observed for concentrations above 10 *μ*M. This introduces a resourceful anticorrelated fluorescence ratio that follows a similar calibration behavior previously shown for the sensors dual-excitation ratio [42]. For concentrations from 200 *μ*M and upwards, an orderly isosbestic point appears at 581.4 ± 0.1 nm (Figure 3(b)). Maximum responses are found at 573 and 596 nm; a calibration curve for that ratio is shown in Figure 3(c) and compared with the nanoparticle free membrane. The 573 nm emission coincides nicely with the FRET overlap (Figure 3(d)). Further, both the 573 and 596 fluorescence wavelengths are red-shifted approximately 10 nm from absorption and fluorescence maximum (Figure 3(d)) of the FRET pair acceptor. This may indicate that the 573 and 596 nm are interconnected transitions that are symmetrically located in the spectra. Analyzing the maximum response wavelengths shows that more photons are transferred to the longer wavelength than are removed from the shorter wavelength emission (Figures 3(b) and 3(c)). These results suggest that there is a Förster-type energy transfer in this system and not simply scattering enhancement, since scattering enhancement would suffer significantly from the lost incident field (donor emission decreases due to solvatochromism) and only minor enhancement could be achieved by approaching the metal surface. A hypothesis is that the proximity of a decoupled dye and the central axis of an imagined plasmophore field sphere (Figure 1) determines the efficiency of energy transfer to the acceptor. Due to plasmophore decoupling and lack of plasmophore/metal absorption at the longer wavelength, the coextracted dye molecule can along the central axis act as an ultimate acceptor and emit the longer wavelength. This might be viewed as a transient and oscillating charge separation within the plasmophore complex, and when the coextracted MC540 molecule approaches the central axis of the plasmophore field, it “short-circuits” the field by resonance and lower ground-state energy. For analytical sensitivity this plasmon-induced ratio will be of primary importance since it has been shown that, for low (nM) concentrations, this sensor type is exclusively responding with the plasmon active Ex511/Em570 nm wavelength pair [42]. Therefore, a significant improvement in sensitivity is expected for this single-excitation dual-emission sensing scheme. Further, this dual-plasmon-connected fluorescence ratio will be more stable and better for ratiometric normalization since both wavelengths are dependent on the same physical feature.

 For building sensor systems with, for example, a single light-emitting diode (LED) and a digital RGB camera as detector, the plasmon-induced single-excitation dual-detection scheme will simplify to a great extent. A real-time ratio will be possible to record, with no filter wheels or multiple light sources/detectors. Also, a single LED is warranted for the benefits of ratiometric measurements, for example, cancellation of light intensity fluctuations.

 Why not simply incorporate a separate acceptor within the ether phase? This will have at least three crucial drawbacks for sensing but might be effective for energy assimilation and charge transfer in Grätzel-type solar cells [45]. The first sensing drawback is that the lipophilic dye will evenly be distributed in the entire ether droplet and no intricate nanoscale organization will transpire. Sensing drawback number two is a consequence from the first: there will be no FRET acceptor dynamics which will diminish most of the signal. The third drawback is that the ratio will be dependent on two fluorophores which eliminate much of the advantage that ratiometric measurements provides; that is, the two fluorophores will likely have quite different leaching and photo bleaching properties.

 The photophysics of merocyanines (e.g., MC540) and the possible utilization in dye-sensitized solar cells [45] have been extensively studied [46]. However, with a low lying triplet state, photodegradation can be a serious problem [46]. Hence, a triplet formation protection that the dynamic surface plasmon coupled system presented in this work offers is of interest. Particularly since the systems intrinsic nanoscale dynamics can both mimic and assist [47] the light-absorbing and photoprotective mechanisms of natural photosynthesis, where the system is able to switch between different energy transfer paths by external stimuli (here ammonium occurrence). Potentially these types of plasmonics can be utilized for thin film engineering [48] to make solar cells more effective and stable, by a broader and more regulated absorption.

 An important detail to investigate for further development of this sensing system is the intrinsic fluorescence lifetimes. This will not only provide important information about the interactions and energy transfer processes but the dynamic plasmonics may also be ideal for highly sensitive lifetime-based sensing [49]. Another pleasant analytical feature of this sensing mechanism that warrants further research is the versatility. In principle it is utilizable for any cation simply by changing the ionophore. An accessible list of some ions with commercially available ionophores can be found on page 26 of [50].

## 4. Conclusions

A resourceful nanoparticle-induced single-excitation dual-emission fluorescence ratio was demonstrated. Plasmonics and dye nanoscale dynamics were attributed to this FRET-type charge transfer. The mechanism can be used for enhanced and simplified cation sensors. The sensing scheme can directly be utilized for all cations with an ionophore (40+ commercially available).
